# Magnetic navigation system for percutaneous coronary intervention: A meta-analysis

**DOI:** 10.1097/MD.0000000000004216

**Published:** 2016-07-22

**Authors:** Zhiyong Qi, Bangwei Wu, Xinping Luo, Jun Zhu, Haiming Shi, Bo Jin

**Affiliations:** Department of Cardiology, Huashan Hospital, Fudan University, Shanghai, China.

**Keywords:** coronary artery disease, magnetic navigation system, meta-analysis, percutaneous coronary intervention

## Abstract

**Background:**

Magnetic navigation system (MNS) allows calculation of the vessel coordinates in real space within the patient's chest for percutaneous coronary intervention (PCI). However, its impact on the procedural parameters and clinical outcomes is still a matter of debate. To derive a more precise estimation of the relationship, a meta-analysis was performed.

**Methods and Results:**

Studies exploring the advantages of MNS were identified in English-language articles by search of Medline, Web of Science, and Cochrane Library Databases (inception to October 2015). A standardized protocol was used to extract details on study design, region origin, demographic data, lesion type, and clinical outcomes. The main outcome measures were contrast consumption, procedural success rate, contrast used for wire crossing, procedure time to cross the lesions, and the fluoroscopy time fluoroscopy time. A total of 12 clinical trials involving 2174 patients were included for analysis (902 patients in the magnetic PCI group and 1272 in the conventional PCI group). Overall, contrast consumption was decreased by 40.45 mL (95% confidence interval [CI] −70.98 to −9.92, *P* = 0.009) in magnetic PCI group compared with control group. In addition, magnetic PCI was associated with significantly decreasing procedural time by 2.17 minutes (95% CI −3.91 to −0.44, *P* = 0.01) and the total fluoroscopy time was significantly decreased by 1.43 minutes (95% CI −2.29 to −0.57, *P* = 0.001) in magnetic PCI group. However, procedural success rate, contrast used for wire crossing, procedure time to cross the lesions, and the fluoroscopy time to cross the lesions demonstrated that no statistically difference was observed between 2 groups.

**Conclusion:**

The present meta-analysis indicated an improvement of overall contrast consumption, total procedural time, and fluoroscopy time in magnetic PCI group. However, no significant advantages were observed associated with procedural success rate.

## 1 Introduction

Percutaneous coronary intervention (PCI) has become the preferred option for the treatment of coronary artery disease (CAD). Although the general success rates of PCI are excellent, the complexity of the coronary artery lesions has also increased markedly, such as stenosis in diffusely diseased vessels, as well as chronic total occlusions, in these lesions, guidewire is always very difficult to pass and can often lead to dissection or perforation of the vessel wall, which is life-threatening. For these severe lesions, the appropriate shape at the tip of the guidewire and the careful navigation in the proper direction to cross the lesion are the key to success.

Recently, a magnetic navigation system (MNS) has been developed, which is an innovative technology that can accurately steer the positioning of a guidewire or a catheter in vivo.^[1]^ It uses 2 computer-controlled magnets to produce an adjustable magnetic field to precisely and actively deflect a tip-mounted magnet on an angioplasty wire, this technology has already been accepted for electrophysiology.^[2–5]^ In recent years, it has been used in PCI, several studies have reported that magnetic percutaneous coronary intervention (MPCI) may improve procedural parameters and clinical outcomes for the treatment of CAD. However, the limited benefits and small sample size of the individual studies have been recently challenged. To derive a more precise estimation of the relationship, a meta-analysis was performed.

## 2 Methods

### 2.1 Study search strategy

This study was carried out and reported in agreement with the PRISMA guidelines for systematic reviews and meta-analyses. Each study was approved by the respective Institutional Ethics Committee. All patients gave written informed consent prior to study inclusion. Studies exploring the advantages of MNS technology were identified in English-language articles by search of Medline, Web of Science, and Cochrane Library Databases (inception to October 2015). We combined search terms for CAD, PCI, and magnetic navigation.

### 2.2 Inclusion criteria

We independently evaluated identified articles for eligibility on the basis of the following inclusion criteria: target population (CAD patients undergoing PCI), intervention (studies of receiving MPCI), and outcomes (test the procedural parameters and clinical outcomes in target population). When the studies were duplicated or overlapped, we included the most recently published studies in the final analysis.

### 2.3 Data extraction

Two reviewers (Qi and Wu) independently extracted data from all eligible studies fulfilling inclusion criteria. If these 2 authors could not reach a consensus, another author (Luo) was consulted to resolve the dispute and a final decision was made by the majority of the votes. A standardized protocol with predefined criteria was used to extract details on study design, region origin, demographic data, lesion type, and clinical outcomes. The main outcome measures were contrast consumption, procedural success rate, contrast used for wire crossing, procedure time to cross the lesions, and the fluoroscopy time in the meta-analysis.

### 2.4 Statistical methods

Cochrane Collaboration meta-analysis review methodology was used for this study. Continuous variables with normal distribution were presented as mean ± standard deviation (SD). Some data were median and 25th, 75th percentiles range, which were used to estimate mean and SD through the following formula: SD ≈ norm interquartile range = (P75–P25)/0.7413 (interquartile range, P75: 75th percentile, P25: 25th percentile).^[6]^ The presence of heterogeneity across studies was evaluated, *P*-value less than 0.10 for the Q test was considered significant for the presence of statistical heterogeneity, so the overall effect estimate was calculated by the random-effect model. Otherwise, the fixed-effect model was used.^[7]^ All statistical tests were performed with RevMan version 4.2.2 available free from Cochrane Collaboration (http://www.cochrane.org/cochrane/hbook/htm).

## 3 Results

### 3.1 Study identification

**Figure 1:**
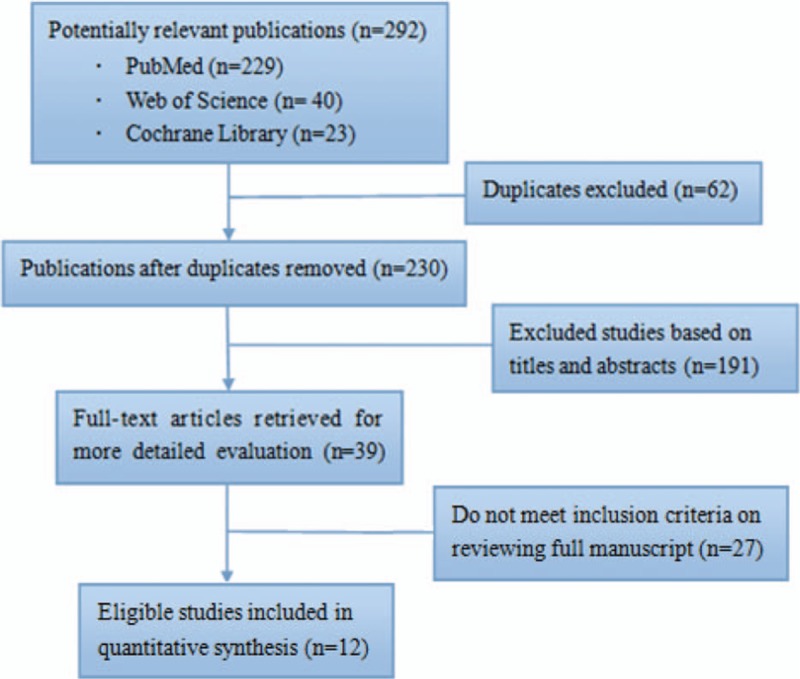
Flow chart of study selection.

The detailed process of study selection was shown in Fig. 1. A total of 292 unique citations were identified by our search strategy. After the initial screening, 39 potentially relevant articles for further review; among these, 27 articles were excluded according to the inclusion criteria. Overall, 12 studies involving 2174 patients were included in the meta-analysis (902 patients in the MPCI group and 1272 in the conventional percutaneous coronary intervention [CPCI] group).^[8–19]^

### 3.2 Study characteristics

Table 1 presents the characteristics of the 12 clinical trials published between 2005 and 2015. Baseline characteristics of the 2 study groups were well balanced with respect to baseline features. In the MPCI group, MNS was used, meanwhile, conventional interventional technologies were used in the CPCI group.

**Table 1:**
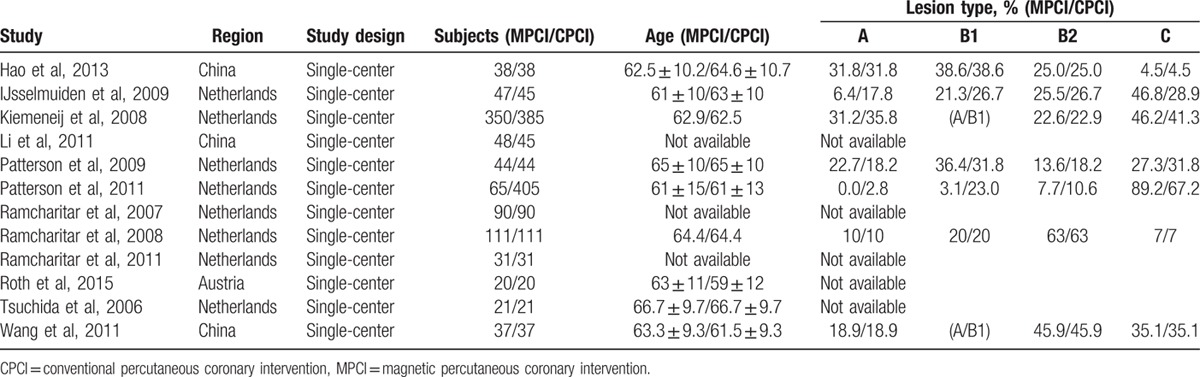
Characteristics of clinical trials reporting magnetic navigation system for percutaneous coronary intervention.

### 3.3 Procedural parameters

**Figure 2:**
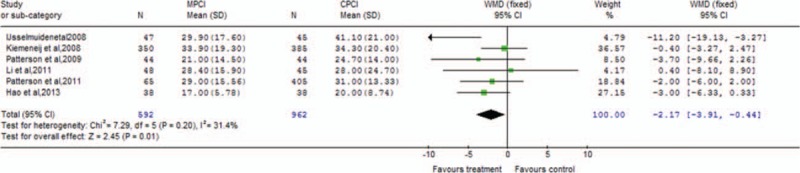
Forest plot of procedural time meta-analysis evaluating MPCI for CAD compared with CPCI (WMD −2.17; 95% CI −3.91 to −0.44; *P* = 0.01). CAD = coronary artery disease, CI = confidence interval, CPCI = conventional percutaneous coronary intervention, MPCI = magnetic percutaneous coronary intervention, WMD = weighted mean difference.

**Figure 3:**
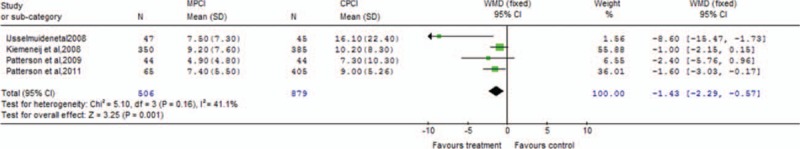
Forest plot of total fluoroscopy time meta-analysis evaluating MPCI for CAD compared with CPCI (WMD −1.43; 95% CI −2.29 to −0.57; *P* = 0.001). CAD = coronary artery disease, CI = confidence interval, CPCI = conventional percutaneous coronary intervention, MPCI = magnetic percutaneous coronary intervention, WMD = weighted mean difference.

**Figure 4:**
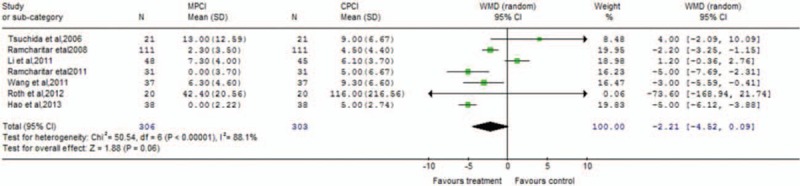
Forest plot of contrast used for wire crossing meta-analysis evaluating MPCI for CAD compared with CPCI (WMD −2.21; 95% CI −4.52 to 0.09; *P* = 0.06). CAD = coronary artery disease, CI = confidence interval, CPCI = conventional percutaneous coronary intervention, MPCI = magnetic percutaneous coronary intervention, WMD = weighted mean difference.

**Figure 5:**
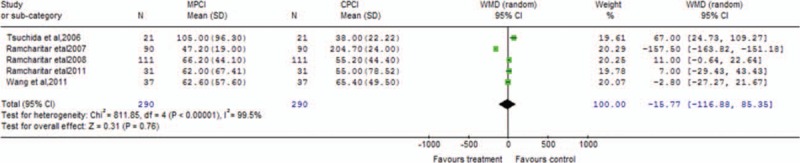
Forest plot of the fluoroscopy time to cross the lesions meta-analysis evaluating MPCI for CAD compared with CPCI (WMD −15.77; 95% CI −116.88–85.35; *P* = 0.76). CAD = coronary artery disease, CPCI = conventional percutaneous coronary intervention, MPCI = magnetic percutaneous coronary intervention, WMD = weighted mean difference.

Table 2 presents the main results of pooled weighted mean differences (WMDs) and odds ratios (ORs) in the meta-analysis. The procedural parameters mainly include total contrast consumption, total procedural time, total fluoroscopy time, contrast used for wire crossing, number of balloons, stents, and wires used per patient, procedure time to cross the lesions, and fluoroscopy time to cross the lesions. Data on total contrast consumption were available in 5 clinical trials, MPCI technology was associated with significantly decreased total contrast consumption (WMD −40.45; 95% confidence interval [CI] −70.98 to −9.92; *P* = 0.009). An analysis of procedural time and total fluoroscopy time indicated that MPCI can advantageously reduce the total procedural time (WMD −2.17; 95% CI −3.91 to −0.44; *P* = 0.01; Fig. 2) and total fluoroscopy time (WMD −1.43; 95% CI −2.29 to −0.57; *P* = 0.001; Fig. 3). However, the WMD estimate indicated that MPCI did not significantly decrease contrast used for wire crossing compared with the control group (WMD −2.21; 95% CI −4.52–0.09; *P* = 0.06; Fig. 4), as well as the number of balloons used per patient (WMD 0.03; 95% CI −0.18–0.24; *P* = 0.80), the number of stents implanted per patient (WMD −0.11; 95% CI −0.39–0.17; *P* = 0.44), the number of wires used per patient (WMD 0.05; 95% CI −0.23–0.32; *P* = 0.73), the procedure time to cross the lesions (WMD −17.95; 95% CI −76.90–41.01; *P* = 0.55), and the fluoroscopy time to cross the lesions (WMD −15.77; 95% CI −116.88–85.35; *P* = 0.76; Fig. 5).

**Table 2:**
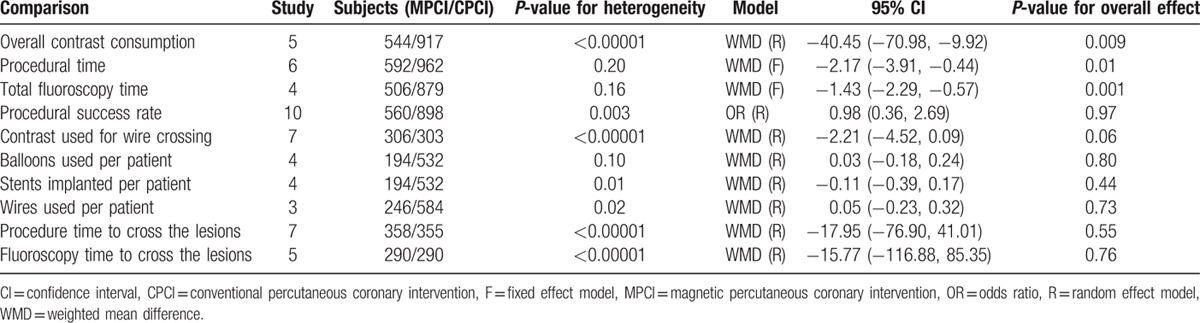
Summary of the meta-analysis of magnetic navigation system for percutaneous coronary intervention.

### 3.4 Clinical outcomes

**Figure 6:**
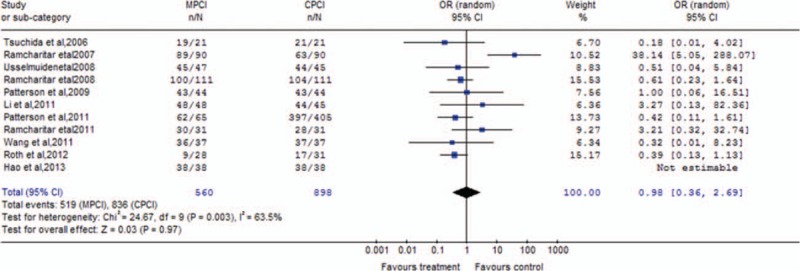
Cumulative OR estimate of procedural success rate in the MPCI group when compared with the control group (OR 0.98; 95% CI 0.36 to 2.69; *P* = 0.97). CI = confidence interval, MPCI = magnetic percutaneous coronary intervention, OR = odds ratio.

As shown in Fig. 6, an efficacy analysis of procedural success rate demonstrated that no statistically difference was observed between MPCI group and CPCI group (OR 0.98; 95% CI 0.36–2.69; *P* = 0.97).

### 3.5 Sensitivity analysis

A single study involved in the meta-analysis was deleted each time to reflect the influence of the individual dataset to the pooled WMDs or ORs, and the corresponding WMDs or ORs were not statistically altered, indicating that our results were statistically robust.

### 3.6 Publication bias

Begg funnel plot and Egger test were performed to access the publication bias of literatures. The funnel-plot analysis indicated that no significant publication bias was detected to influence the results of this meta-analysis.

## 4 Discussion

MNS is a developed technology for interventional cardiology, it is logical to consider its advantage on procedural parameters in PCI. As a promising technology, MNS can orient the tip of the guidewire in the right direction rather than merely pushing it forward. The application of the MNS can ensure to decrease the impairment to coronary artery wall in complex lesions.^[20,21]^ In addition, the deflection force that the external magnetic field can exert is lower than the mechanical push force of conventional guidewire.^[22]^ The important advantage of MPCI is that it can result in a substantial reduction in X-ray to cardiologists as well as a potential decrease in contrast induced nephropathy in high risk patients.^[23]^

Although MPCI has many advantages, the interventional community has been slow to adopt MPCI, maybe there are several reasons in the following: there is rich experience for CPCI, and the performance of manually navigated guidewire has improved exponentially; MPCI takes extra time for assigning the desired vector of the tip of the guidewire using the current user interface; the rigid magnetic wire tip is difficult to cross the sharply angulated turns that are narrow and constrained, and MPCI requires extra attention in order to avoid such problems as magnetic wire lock;^[24]^ MNS is highly expensive and there is also additional cost for staff training; the “ocular-hand coordination” seems currently difficult to be achieved because of the delay in responsiveness in the MNS hardware; and there is a steep learning curve that must be achieved by cardiologists, nurses and skilled technical staffs.

Studies to explore the pros and the cons of the MPCI were carried out in many centers, but the results were controversial in different types of coronary artery lesions, such as simple coronary lesions, complex and distal lesions, bifurcation lesions, and so on. Our meta-analysis indicated an improvement of overall contrast consumption, total procedural time, and total fluoroscopy time in MPCI group compared with CPCI group. Although no significant advantages were observed associated with procedural success rate, contrast used for wire crossing, number of balloons used per patient, number of stents implanted per patient, number of wires used per patient, the procedure time to cross the lesions, and the fluoroscopy time to cross the lesions, we think that MPCI can improve these procedural parameters and clinical outcomes with the increase of the operators’ experience in the future. However, more randomized controlled trials are needed in treating different types of coronary artery lesions.

The pooled results that we report need to be interpreted with some caution. First, the meta-analysis was not performed on individual patient data, so the data were partly extracted from 12 clinical trials for analysis. Second, the publication bias, favoring the publication of positive studies, cannot be excluded. Third, the potential heterogeneity among trials, due to varying inclusion criteria and different levels of experience among operators, also cannot be excluded.

In summary, the present meta-analysis indicates an improvement of overall contrast consumption, total procedural time, and total fluoroscopy time in MPCI group compared with CPCI group. However, no significant advantages were observed associated with procedural success rate, contrast used for wire crossing, the procedure time, and the fluoroscopy time to cross the lesions. Given the relatively small sample size, larger scale prospectively designed randomized double-blind trials should be carried out to clarify the potential benefits of MPCI for CAD.
